# Diagnosis of Dental Fluorosis Using Micro-Raman Spectroscopy Applying a Principal Component-Linear Discriminant Analysis

**DOI:** 10.3390/ijerph182010572

**Published:** 2021-10-09

**Authors:** Marco Antonio Zepeda-Zepeda, Michel Picquart, María Esther Irigoyen-Camacho, Adriana Marcela Mejía-Gózalez

**Affiliations:** 1 Physics Department, Universidad Autónoma Metropolitana (UAM) Unidad Iztapalapa, Mexico City 09340, Mexico; 2 Health Care Department, Universidad Autónoma Metropolitana (UAM) Unidad Xochimilco, Mexico City 04960, Mexico; mp@xanum.uam.mx (M.P.); meirigo@correo.xoc.uam.mx (M.E.I.-C.); 3 Oral Health Sub-Direction, Centro Nacional de Programas Preventivos y Control de Enfermedades, Secretaría de Salud, Mexico City 11800, Mexico; adriana.mejia@salud.gob.mx

**Keywords:** dental fluorosis, Raman spectroscopy, principal component analysis, discriminant analysis

## Abstract

Dental fluorosis is an irreversible condition caused by excessive fluoride consumption during tooth formation and is considered a public health problem in several world regions. The objective of this study was to evaluate the capability of micro-Raman spectroscopy to classify teeth of different fluorosis severities, applying principal component analysis and linear discriminant analysis (PCA-LDA), and estimate the model cross-validation accuracy. Forty teeth of different fluorosis severities and a control group were analyzed. Ten spectra were captured from each tooth and a total of 400 micro-Raman spectra were acquired in the wavenumber range of 250 to 1200 cm^−1^, including the bands corresponding to stretching and bending internal vibrational modes ν_1_, ν_2_, ν_3_, and ν_4_ (PO_4_^3−^). From the analysis of the micro-Raman spectra an increase in B-type carbonate ion substitution into the phosphate site of the hydroxyapatite as fluorosis severity increases was identified. The PCA-LDA model showed a sensitivity and specificity higher than 94% and 93% for the different fluorosis severity groups, respectively. The cross-validation accuracy was higher than 90%. Micro-Raman spectroscopy combined with PCA-LDA provides an adequate tool for the diagnosis of fluorosis severity. This is a non-invasive and non-destructive technique with promising applications in clinical and epidemiological fields.

## 1. Introduction

Dental fluorosis is an irreversible condition characterized by the hypomineralization of the dental structure that occurs during tooth formation. Children chronically exposed to high levels of fluoride in the first six years of life will develop dental fluorosis in the permanent dentition [1]. This condition affects millions of people worldwide. The mild form of fluorosis is the most prevalent [2]. The main source of fluoride is groundwater, which is frequently of geological origin. Several fluoride belts have been identified: one running through Turkey, Iraq, Iran, Afghanistan, India, Northern China, and Thailand, and another one stretching from Syria through Jordan, Egypt, Libya, Algeria, Sudan, and Kenya [2]. In the Americas, some regions have high fluoride content in groundwater, including some southern areas of the United States and Northern Mexico [3,4].

An increase in dental fluorosis has been identified in several countries [5,6]. This condition is considered a late sign of the chronic consumption of fluoride, and it is a sensitive indicator of high fluoride exposure during infancy [7]. Fluoride can provide protection against dental caries when used properly. In fact, fluoridation of drinking water was designated by The Centers for Disease Control and Prevention in the USA, as one of the 10 most important public health achievements of the 20th century, due to its benefits in dental caries prevention shown since the 1960s [8]. Additionally, salt fluoridation has also demonstrated its ability to prevent and control dental caries [9]. The very mild and mild categories of fluorosis have been associated with a low risk of dental caries [10]. Conversely, high fluorosis severity is associated with higher caries experience and lower oral-health-related quality of life [4,11].

The external structure of teeth is dental enamel, which is the hardest tissue in the human body. Dental enamel has a high mineral content (95–98%) and it structured in long and parallel hydroxyapatite-type crystals rods with an interlaying organic matrix (0.2–2.0%) [12,13]. Hydroxyl calcium phosphate is the main mineral constituent of the teeth (OHAp), represented by Ca_5_(PO_4_)_3_(OH). The general structure of the enamel crystals is a hexagonal system, with a symmetry that corresponds to the spatial group P6_3_/m. OHAp belongs to a large group of minerals called apatites. Their chemical composition is Ca_10_(PO_4_)_6_X_2_, where X can be OH (hydroxyapatite, OHAp), fluoride F (fluorapatite, FAp), chloride Cl (chlorapatite, ClAp) [14], etc. Substitution can occur at multiple sites of the hydroxyapatite crystals. In A-type carbonate substitution, carbonate ions can substitute into the hydroxyl site, and in B-type carbonate substitution, carbonate ions can substitute into the phosphate site. At low concentrations, various minerals (Mg, Na, Si, and Sr) and fluoride can substitute into the apatite lattice [15,16]

Teeth with mild dental fluorosis present white horizontal lines on the enamel; as the level of fluorosis increases, these lines are more pronounced and confluent, becoming opacities that can be white or brown in color. Teeth with severe fluorosis present post eruptive loss of enamel and the entire appearance of the tooth could deteriorate [17]. The clinical assessment of dental fluorosis severity can be difficult, but it is important to accurately evaluate fluorosis to detect geographical regions with an increased risk of dental fluorosis and to identify changes in the time trends of fluorosis. Preventive programs of dental caries and fluorosis would benefit from the accurate assessment of this condition.

Raman spectroscopy is a noninvasive and nondestructive optical scattering technique used to analyze the materials’ molecular structure. The Raman effect occurs when a light beam hits the surface of a specimen, and some of the scattered photons undergo an energy change. Each molecule has characteristic vibrational modes, constituting a fingerprint of the molecule. Raman spectroscopy is used to capture these vibrational modes and express them as a series of peaks in the spectrum that characterize a specific molecule or tissue. Raman spectroscopy has been used in the study of developmental anomalies of dentition, such as dental fluorosis and molar-incisor hypomineralization or oral disease such as dental caries [13,18,19]. A study using Raman spectroscopy for chemically mapping the enamel and dentin in teeth with molar–incisor hypomineralization found that the severity of the lesion is associated with disorders in the hydroxyapatite crystals and the presence of organic species. The authors concluded that Raman spectroscopy could be a valuable instrument in the study of changes in enamel-related hypomineralization defects [13]. 

The analysis of Raman spectra generally involves multiple variables, and compressed data statistics techniques are applied. Principal component analysis (PCA) has successfully been used for reducing Raman spectra dimensionality [20]. The combination of PCA with linear discriminant analysis (PCA-LDA) is a powerful statistical tool for screening and data analysis in health sciences. This technique allows for the building of machine learning models, which can be used to classify new samples [21,22,23,24]. A better understanding of the crystal structure of fluorotic enamel and its relationship with clinical presentation may contribute to improving the diagnosis of this condition. 

Raman spectroscopy has been extensively used to analyze the molecular structure, chemical composition, and intermolecular interactions of several biomaterials [25,26,27]. However, this technique has seldom been used in the study of changes produced by fluoride in dental tissues [18,28]. As such, the objective of this study was to apply micro-Raman spectroscopy to classify teeth with different fluorosis severities using the PCA-LDA model and to estimate its cross-validation accuracy for the diagnosis of dental fluorosis.

## 2. Materials and Methods

### 2.1. Sample

Teeth were selected from the northern-central area of Mexico, where the range of water fluoride was 0.1 to 4 ppm [29]. The samples used in this study were obtained from teeth that were extracted at the Ministry of Health Public Dental Clinics and donated by patients; the age range of the patients was from 18 to 35 years. Twelve premolars and twenty-eight molars were included in the study sample. The reasons for extraction of the teeth were orthodontic treatment, prosthesis placement, and dental caries. No restorations were present in the teeth. In this study, the buccal surfaces were selected for fluorosis assessment. Teeth with stains or fractures on the buccal surface were excluded. Special precautions were taken to select teeth without non-cavitated (white spots) to extensive cavitated caries or lesions on these surfaces, applying the ICDAS index. (ICDAS 2009) [30]. The Russell’s criteria, adapted by Pendrys, were applied for differential diagnosis of milder forms of enamel fluorosis from non-fluoride enamel opacities [31].

The transportation guidelines of the Mexican Ministry of Health for biological specimens were followed [32]. The protocol was approved by the Scientific Committee of the Doctoral Program in Physics, Universidad Autónoma Metropolitana-Iztapalapa and its ethical aspects were reviewed and approved (CBI.AP.962.2015).

### 2.2. Fluorosis Classification

Dental fluorosis was assessed using the Thylstrup and Fejerskov Index (TFI). This index has 10 scores related to the histological features behind each individual fluorosis category. The initial levels are associated with horizontal narrow lines following the perikymata; moderate forms of this condition show the entire tooth surface as opaque or chalky white with small areas of pitting with loss of enamel <2 mm. Severe forms have confluent pitting derived from loss of horizontal bands of enamel. In the highest category there is loss of the main parts of the enamel, noticeably changing the appearance of the tooth [17]. The TFI was recorded independently by two trained and calibrated examiners (MEI and AMG) who had participated in the criteria standardization process of the National Oral Survey of Caries and Fluorosis in Mexico. When disagreement between examiners occurred during the classification of fluorosis severity, a discussion took place and agreement was reached. The teeth examined were classified into four groups: sound enamel (TFI = 0), mild (TFI = 1–3), moderate (TFI = 4–6), and severe (TFI = 7–9). A total of 40 erupted posterior teeth were included, 10 in each fluorosis category group, and 10 with sound enamel, as negative controls. 

### 2.3. Sample Preparation 

After extraction, teeth were stored in deionized water saturated with 0.1% thymol until taken to the laboratory for processing. The samples were washed with deionized water to eliminate organic material and grease from the teeth and each one was sonicated in a deionized water bath for 30 min (Ultrasonic Cleaner with Timer, 8890R-MYH model, Cole Parmer, Vernon Hills, IL, USA). The teeth were air-dried (Dust-Off Compressed Air, Edmund Optics, Barrington, NJ, USA) before Raman spectra acquisition.

### 2.4. Micro-Raman Spectroscopy

**Figure 1:**
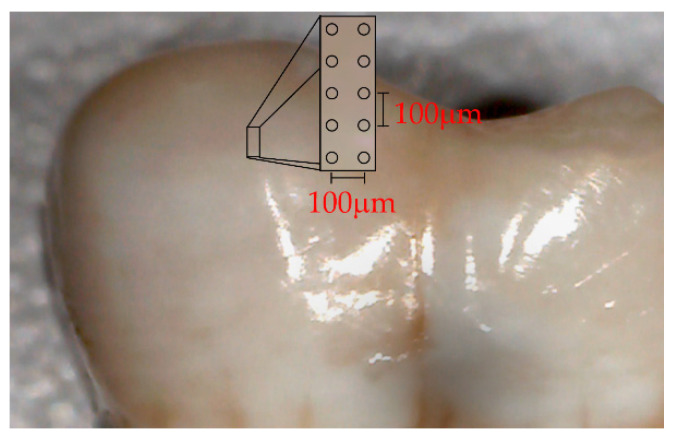
Photograph depicting a rectangular area used for Raman spectra acquisition under a digital microscope.

Raman spectra were captured on a T64000 triple spectrometer (Horiba Jobin Yvon, Edison, NJ, USA) using a green laser at 532.1 nm (Laser Quantum DPSS, Ventus 532 + mpc 6000, Laser Quantum, Stockport, Cheshire, UK) and a confocal microscope with a 100× objective (Olympus, 20097 Hamburg, Germany). Each spectrum was formed by 10 accumulations of 60 s. The spectra were acquired from representative tooth enamel fluorosis regions, mapped on the sagittal axis of the buccal surface, forming approximately a perpendicular angle with the perikymata lines. Ten different points were chosen with a 100 µm gap between each, parallel to the longitudinal axis of the tooth sampled from a rectangular micro-area of 400 µm x 100 µm, as illustrated in Figure 1 (Digital Microscope AMH DUT Dino Lite Dentiscope, Zervex, Singapore). The power of the beam was 20 mW. The Raman equipment was calibrated using a silicon semiconductor and a 520 cm^−1^ peak was selected for this purpose. LabSpec software (Horiba, Kyoto, Japan) was used for setting control and spectra acquisition. The spectral resolution was 0.1 cm^−1^.

All the row spectra were pre-processed to remove differences related to baseline and scale. Smoothing was performed with a local polynomial regression fitting function and concave rubber band baseline correction was performed using hyperSpecJSS software [33] and R software (R Core Team 2017), R language, and environment for statistical computing (R Foundation for Statistical Computing, Vienna, Austria). Additionally, each spectrum was normalized by the total area of the Raman spectrum in the wavenumber range of 250 to 1200 cm^−1^. From the 40 teeth sampled, a total of 400 Raman spectra were collected: 100 spectra from 10 sound teeth, and 300 from the 30 teeth with fluorosis.

The Raman spectrum of OHAp is strongly dominated by the internal vibrational modes of the phosphate group [34]; the PO_4_^3−^ bands are shifted in relation to free ion normal mode due to the crystalline field effect [35]. Penel et al. provided the band wavenumber and associated assignment of the main dental enamel bands as follows: ν1 (PO_4_^3−^) at 959 cm^−1^, ν2 (PO_4_^3−^) at 433 and 450 cm^−1^, ν3 (PO_4_^3−^) at 1026, 1043 and 1071 cm^−1^, and ν4 (PO_4_^3−^) at 579, 588 and 608 cm^−1^. The band at 1071 cm^−1^ is representative of the B-type carbonate substitution in the hydroxyapatite [14]. The region selected in this study was 250–1200 cm^−1^ for including these bands.

### 2.5. Statistical Analysis

The Raman spectra means were obtained for the sound, mild, moderate, and severe categories. Raman spectra provide multidimensional data (this is the case when the number of variates is larger than the number of specimens observed). ANOVA was used to test differences between means of peak heights and between band integral ratios among fluorosis categories. LDA was applied for classification of multiple groups. Prior to the LDA, a reduction in the spectrum dimensions was achieved using PCA. This analysis was used to transform the original information of the spectra into a reduced new set of variables in the PCA space, considering the variables’ inter-correlations. A quadratic discriminant analysis (QDA) model (PCA-QDA) was also fitted for the discrimination of fluorosis severity. Statistical significance was set at α < 0.05. The statistical analysis was performed using R packages (R Foundation for Statistical Computing, Vienna, Austria). 

## 3. Results

**Figure 2:**
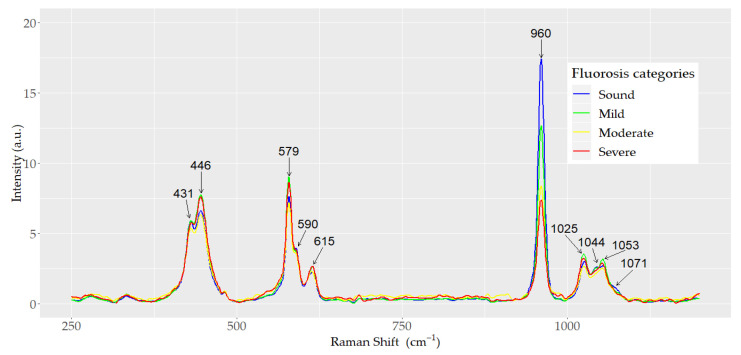
Mean normalized Raman spectra of sound, mild, moderate, and severe dental fluorosis categories.

A total of 400 spectra of dental enamel were analyzed. The spectra were acquired from 40 teeth: 10 teeth from each of the three dental fluorosis categories studied (mild, moderate, and severe) and 10 sound controls. Ten spectra were obtained from each tooth. The mean normalized Raman spectra for each dental fluorosis category and control samples are depicted in Figure 2. The internal vibrational modes of the phosphate ion (PO_4_^3−^) in the hydroxyapatite are presented. All the spectra exhibited the same general pattern bands, but differences in peak intensity were observed among the dental fluorosis categories. The highest peak corresponding to the symmetric stretching vibrational mode ν_1_ (PO_4_^3−^) was present at 960 cm^−1^. The height of this peak was inversely associated with fluorosis severity, as severity of fluorosis increased as the height of the peak decreased (*p* < 0.001). The peaks associated with the bending vibrational mode ν_2_ (PO_4_^3−^) were observed at 431 and 446 cm^−1^. Peaks corresponding to the asymmetric stretching vibrational mode ν_3_ (PO_4_^3−^) were observed at 1025 and 1053 cm^−1^ with a shoulder at 1044 cm^−1^. The peaks and shoulder corresponding to the bending vibrational mode ν_4_ (PO_4_^3−^) were detected at 579, 590, and 615 cm^−1^. The characteristic symmetric vibrational mode ν_1_ (CO_3_^2−^) was observed at 1071 cm^−1^. No significant differences were found in the height of the internal vibrational mode ν_2_ (PO_4_^3−^) between the mild and healthy categories (*p* > 0.05).

**Figure 3:**
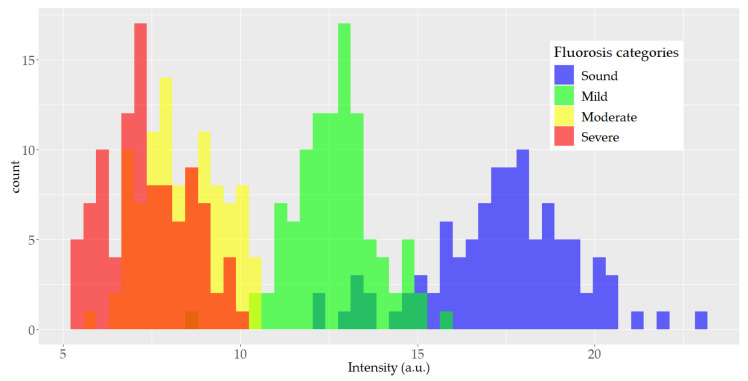
Histogram of the height distribution of the 960 cm^−1^ Raman spectrum band by fluorosis category (100 spectra in each category).

Figure 3 depicts the histogram of the peak height at the 960 cm^−1^ band associated with vibrational mode ν_1_ (PO_4_^3−^). A large overlapping between the moderate and severe histograms was detected and smaller overlapping between the other categories was observed.

ANOVA was performed to compare the mean band integral ν_1_ (CO_3_^2−^)/ν_1_ (PO_4_^3−^) ratio among the dental fluorosis categories analyzed. The results indicated a significant difference in this band integral ratio; the mean of this ratio increased as the fluorosis category increased (*p* < 0.001). Tukey’s test showed significant differences in this ratio between all pairs of fluorosis severity categories (*p* < 0.001) except between sound and mild groups (*p* = 0.350).

### 3.1. Principal Components Analysis and Linear Discriminant Analysis

**Figure 4:**
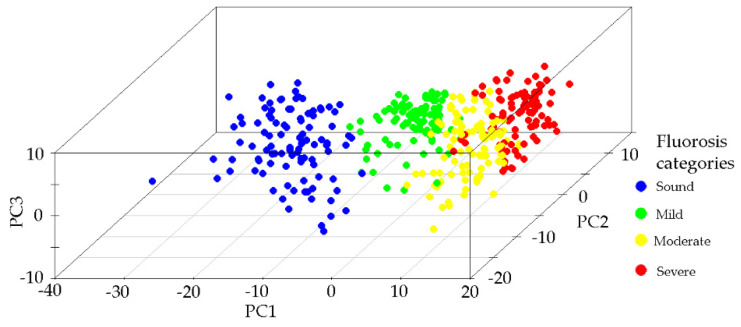
Principal component analysis (PCA). Scatter plot in the 3D PCA space of the Raman spectra according to the fluorosis category.

A PCA model was built using the normalized Raman spectra. The spectra region studied ranged from 250 to 1200 cm^−1^. PCA was used to reduce the dimensionality of the complex Raman spectra and to create a new space (PCA space) with the orthogonal axis defined by the eigenvectors. The first principal component (PC1) explained 51.27% of the variance, the second 13.73%, and the third 12.49%. From the fourth to the sixth PCs, the explanation was low, around 1%. The total variance explained by the first three principal components was 77.49%. Figure 4 presents the scatter plot in PCA space where each point corresponds to one spectrum and the clusters observed are related to each of the fluorosis severity categories and the control group. Samples of sound enamel are situated in the negative values of PC1; as severity increases, the points are situated toward the positive values of PC1. PC2 contributed to differentiating the cluster formed by the sound and mild categories. 

**Figure 5:**
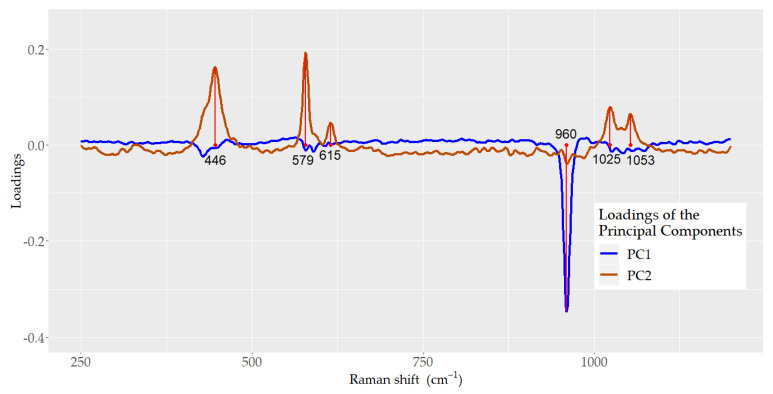
Principal component analysis (PCA). Loadings plot of principal component 1 (PC1) and principal component 2 (PC2) of the Raman spectra data.

Figure 5 depicts the loadings for PC1 and PC2. For PC1, the main loading corresponded to ν_1_ (PO_4_^3−^), situated at 960 cm^−1^, whereas its role in PC2 was minor compared with the loadings of the bands located at 446 and 579, which correspond to the vibrational modes ν_2_ (PO_4_^3−^) and ν_4_ (PO_4_^3−^), respectively, and 1025 and 1053 cm^−1^, which correspond to the ν_3_ (PO_4_^3−^) vibrational mode.

### 3.2. Linear Discriminant Analysis and cross-Validation Analyses

Linear discriminant analysis (LDA) was constructed using the scores obtained from the first three PCs. LDA constructed discriminant functions that allowed us to classify samples based on the probability of belonging to a certain category. To build the model, the spectra data were partitioned into two subsets: the first one was used as the training set to develop the model, and the second one was used to test the model through a cross-validation process. This partition was completed by applying a random stratified sampling method; 70% of the teeth were included in the training set (7 teeth from each category) and 30% of the teeth were included in the test set (3 teeth from each fluorosis category). Therefore, 280 and 120 spectra were used for the training and test sets, respectively.

The first discriminant function (LD1) was:

LD1 = 0.2883(PC1) + 0.1326(PC2) + 0.0392(PC3)

The percentage of the between-class variance explained by the first and second linear discriminant functions were 96.5% and 3.5%, respectively. 

LDA correctly classified 262 spectra, for a 93.58% training accuracy. In the test set, 110 spectra were correctly classified by the model, resulting in a cross-validation accuracy of 91.67%. Table 1 presents the correct classification probabilities of the test sample for the fluorosis categories. A sound tooth had a 0.97 probability of being correctly classified, and a tooth with severe fluorosis had a 0.93 probability of being correctly classified.

**Figure 6:**
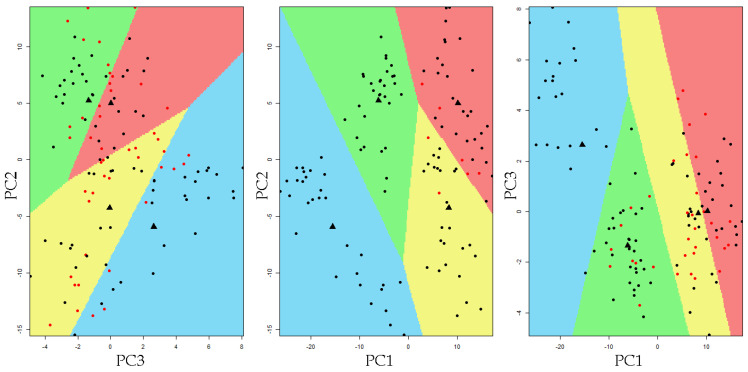
Partition plot resulting from the linear discriminant analysis in the 3D principal component space. The colors set the limits of each classification area defined by the LDA model (blue = sound, green = mild, yellow = moderate, and red = severe). The black marks indicate the sample was correctly classified, while red marks indicate the sample was incorrectly classified by the model. The black dots represent the group mean value.

Figure 6 presents the partition plot in PCA space. Colored regions set the limits of each classification area. The observation that fall into a particular colored region correspond to its LDA-predicted membership. Most of the observations fell into the region corresponding to the clinically defined fluorosis category.

Table 2 presents the specificity, sensitivity, and accuracy of the different fluorosis categories. The highest sensitivity (98.9%) was achieved between fluorosis presence (mild, moderate, or severe) and sound teeth. A 100% specificity was obtained when comparing the severe and moderate categories with the mild and sound group. The comparison of the other categories attained a specificity higher than 94%. Similarly, accuracy was also high. The quadratic discriminant analysis showed a cross-validation accuracy of 90.0%, which was lower than that obtained by LDA.

## 4. Discussion

The findings of this study reveal that dental fluorosis severity can be assessed using micro-Raman spectroscopy and PCA-LDA models. The micro-Raman spectra studied showed that the intensity of the ν_1_ (PO_4_^3−^) peak at 960 cm^−1^ decreased as fluorosis severity increased. This characteristic supports the notion that the 960 cm^−1^ peak could be useful in the identification of teeth with dental fluorosis [36]. However, as depicted in the histogram of the peak height of ν_1_ (PO_4_^3−^), good classification of fluorosis severity is not yet possible since strong overlapping occurs among fluorosis categories, particularly between the moderate and severe fluorosis levels. The intensity of the ν_1_ (PO_4_^3−^) band is produced not only by the chemical composition and structure of the enamel crystals, but also by the specific orientation of the apatite crystals with respect to the incidence beam of light and the angle at which the dispersion light is observed. Consequently, the fluorosis classification based only on this information may produce ambiguous results. This phenomenon has already been reported by Buchwald et al. [37].

In the current study, the peak integral ratio 1071/960 cm^−1^ (carbonate/phosphate ratio) significantly increased as fluorosis severity increased. Consequently, the results indicate that the B-type substitution increases as the severity of fluorosis increases. A previous study using Raman spectroscopy found a significant association between carbonate content, crystallinity, and the mechanical properties of the apatite, such as modulus and hardness. Higher carbonate levels have been associated with lower quality of dental enamel caused by the changes in apatite crystallinity [38]. Crystallinity affects the mechanical properties of dental enamel. Consistently, in a study of the biomechanical properties of irradiated bone, an increased carbonate/phosphate ratio was associated with a decrease in crystallinity compared with non-irradiated controls. Irradiated bone is more brittle and has a higher risk of fractures than healthy bone [39]. The enamel biomechanical properties have implications for treatment options related to tooth restoration design and materials, impacting the longevity of the restorations.

The PCA model fitted in this study showed that the points in 3D PCA space formed clusters according to the level of dental fluorosis, implying that the samples in each category have similar chemical compositions and structures [13]. The PC1 axis allowed the spreading of these clusters in 3D PCA space. The intermediate categories, mild and moderate, were located between the extreme categories. PC2 and PC1 jointly aided to separate the mild category from the sound group in this space. The PCA results reveal that this analysis is adequate for reducing the dimensionality of micro-Raman fluorosis spectra.

The loading of the eigenvectors showed that the vibrational mode ν_1_ (PO_4_^3−^) at 960 cm^−1^ has a dominant role in PC1, but a minimal contribution to PC2, which depends on the internal vibrational modes ν_2_, ν_3_, and ν_4_ (PO_4_^3−^). This information reflects the need to include other vibrational modes besides ν_1_ (PO_4_^3−^) in the analysis of the spectra for dental fluorosis diagnosis.

The linear discriminant analysis used the first three PCs for the construction of discriminant functions. The results showed that the first discriminant function (LD1) explained 96.5% of the between-class variance, providing an adequate separation between fluorosis groups. The partition plot illustrates the acceptable capability of the model to classify teeth with fluorosis. These results favor the application of a quantitative criteria to allocate samples to a fluorosis category according to the key features of their spectra. The cross-validation accuracy was good (91.7%); accordingly, the sensitivity and specificity were high. Another study using micro-Raman spectroscopy and PCA have reported similar results, with specificity and sensitivity values higher than 90% [18].

The test error of the PCA-LDA model was approximately 8%. Chemical substitutions and structural variation in the enamel crystals may contribute to this shortcoming. During tooth formation, the fluorosis process produces changes in protein/mineral interactions with the retention of amelogenins, generating hypomineralized areas. Fluoride increases the mineral precipitation process during tooth formation, and high levels of fluoride produce hypermineralized bands of enamel, which are then followed by hypomineralized bands [40]. A study using an ion-specific fluoride electrode and pH/ion meter to quantify fluoride revealed that fluoride content in the enamel may overlap fluorosis categories [41].

Animal studies demonstrated the occurrence of fluoride-induced fluctuations in calcium concentration in the mineralizing milieu of the teeth. Fluoride causes calcium-traumatic responses in two directions: first, hypermineralization is produced as a result of temporary rapid growth in calcium precipitation; as calcium levels decrease, hypomineralization occurs with a subsequent increase in enamel porosity [1,42,43]. The lack of homogeneity in hydroxyapatite crystal growth observed in dental fluorosis most likely contributed to the misclassification of specimens by PCA-LDA.

In the present study, in addition to the PCA-LDA model, quadratic discriminant analysis (PCA-QDA) was applied. The PCA-QDA model had a cross-validation accuracy of 90%. The quadratic analysis produced slightly lower values compared with PCA-LDA. The results showed that the data could be modeled using a linear model without a loss in its discriminant capacity. Simpler models are favored because they are easier to interpret and to apply to other samples.

Among the strengths of this study is the systematic mapping of the tooth surface sites for spectra acquisition. A carefully standardized process for micro-Raman spectra was performed. All the spectra were pre-processed. To remove differences in baselines, concave rubber band correction was used after smoothing. A general standardization process was followed using the area under the curve in the studied wavelength range.

A total of 400 spectra were acquired. The large number of observations enabled the use of appropriate statistical tools to model the experimental data. Using a machine learning perspective to develop algorithms for the analysis of high-dimensional and multimodal data for predictions, it was possible to build an algorithm to classify teeth with dental fluorosis and test its accuracy using validation techniques [24]. The developed algorithm, the PCA-LDA model, could be applied to other samples. This may be useful for examining the generalizability of the results.

A limitation of the study is that not all tooth surfaces were mapped. All spectra were recorded on the buccal surface of the tooth sample. It is possible that other surfaces, i.e., the occlusal surface, would provide additional data about the differences in crystal orientation among the tooth surface. Erosion and attrition of the enamel could also influence the results. However, the buccal surface was chosen not only considering the aesthetic impact of dental fluorosis and the visibility of this surface but also its accessibility during oral examination. Another limitation was that the gold standard of fluorosis diagnosis is based on clinical criteria according to Thylstrup and Fejerskov [17]; however, two experienced examiners classified each tooth and in the case of disagreement the teeth were reexamined and an agreement on the diagnosis was reached. More micro-Raman spectroscopy studies for the diagnosis of dental fluorosis are required. For example, refining the process of mapping the enamel areas to be observed may help to reduce the misclassification of dental samples. The inclusion of polarized micro-Raman techniques may improve the accuracy of the predictions, as this tool can recognize crystal orientation; however, this method has, so far, been performed only in ex vivo specimens [44].

A difficulty encountered by health professionals in the preparation of examiners to diagnose dental fluorosis is the complexity of the standardization process; this increases the risk of bias. A visual guide showing the different levels of dental fluorosis severity, previously classified by experts and Raman-spectra PCA-LDA models, could be useful for standardization purposes. This may assist with training examiners in the clinical, epidemiological, and research fields.

Fluoride plays a key role in the prevention and control of dental caries. The risk of dental fluorosis has been an issue in preventive programs based on the addition of fluoride to drinking water, salt, milk, and other products. There is a difficult balance between caries prevention and the development of dental fluorosis. The Environmental Protection Agency in the US Institute of Medicine provided a tolerable upper intake level of 0.10 mg fluoride/kg body weight/day; however, this limit has been exceeded in several populations without the appearance of negative effects on dental enamel [45,46]. Conversely, some populations appear to be more sensitive to fluoride than others [47]. An optimum dose is under discussion, but the final test should be based on the results of monitoring programs where both dental caries and dental fluorosis are evaluated [45].

This emphasizes the need for accurate dental fluorosis diagnosis techniques, which would facilitate the surveillance of this developmental defect. This would contribute to the implementation of intervention strategies and to the regulation of public health policies, such as water and salt fluoridation programs.

The future applications of Raman spectroscopy in the clinical setting are promising, considering the recent development of prototypes and some portable commercially available devices. Portable or handheld Raman spectrometers equipped with optical fiber may be used for the mapping of in vivo specimens. In dental surgery, several spectra of a tooth may be acquired to obtain valuable information for the diagnosis of dental fluorosis severity [48].

Fluorosis is a growing public health problem in regions with high groundwater fluoride concentrations [49]. Dental fluorosis may be a marker of the presence of water sources with high fluoride content, which could be used to alert health authorities. Several diseases have been associated with high chronic fluoride exposure [50], and one study found an association between high fluoride exposure and low IQ in children [51]. The availability of accurate, non-destructive, and non-invasive methods for the diagnosis of dental fluorosis is an appropriate step in preventing and controlling severe forms of this condition.

## 5. Conclusions

The analysis of the Raman spectra showed that B-type carbonate ion substitution into the phosphate site of the hydroxyapatite increases as fluorosis severity increases. PCA indicated that ν_1_ (PO_4_^3−^) at 960 cm^−1^ played a dominant role in PC1 and did not contribute to PC2, whereas vibrational modes ν_2_, ν_3_, and ν_4_ (PO_4_^3−^) provided additional information to discriminate between mild and sound fluorosis categories. This emphasizes the importance of considering several bands in the analysis of the spectra, since they improve the identification of fluorosis severity. The PCA-LDA model showed high validation accuracy, sensitivity, and specificity for the identification of dental fluorosis categories. Micro-Raman spectroscopy and PCA-LDA models represent an advance in the diagnosis of dental fluorosis and could be used in different clinical and epidemiological settings.

## Figures and Tables

**Table 1:** Linear discriminant analysis (LDA). Probability of being correctly classified by the LDA model for the dental fluorosis categories.

Fluorosis Categories	Probability
Sound	0.9667
Mild	0.9667
Moderate	0.8000
Severe	0.9333

**Table 2:** Linear discriminant analysis (LDA). Sensitivity, specificity, and accuracy of the LDA model comparing dental fluorosis categories.

Fluorosis Categories	Sensitivity	Specificity	Accuracy
Severe	93.3	94.4	94.2
Severe + Moderate	96.7	100.0	98.3
Severe + Moderate + Mild	98.9	96.7	98.3

## Data Availability

The datasets generated and/or analyzed during the current study are available from the corresponding author upon reasonable request.

## References

[1] Aoba T., Fejerskov O. (2002). Dental fluorosis: Chemistry and biology. Crit. Rev. Oral Biol. Med..

[2] World Health Organization Water Sanitation. Water Related Diseases. Fluorosis. https://www.who.int/water_sanitation_health/diseases-risks/diseases/fluorosis/en/.

[3] Beltrán-Aguilar E.D., Griffin S.O., Lockwood S.A. (2002). Prevalence and Trends in Enamel Fluorosis in the United States from the 1930s to the 1980s. J. Am. Dent. Assoc..

[4] Molina-Frechero N., Nevarez-Rascón M., Nevarez-Rascón A., González-González R., Irigoyen-Camacho M.E., Sánchez-Pérez L., López-Verdin S., Bologna-Molina R. (2017). Impact of Dental Fluorosis, Socioeconomic Status and Self-Perception in Adolescents Exposed to a High Level of Fluoride in Water. Int. J. Environ. Res. Public Health..

[5] Wiener R.C., Shen C., Findley P., Tan X., Sambamoorthi U. (2018). Dental Fluorosis over Time: A Comparison of National Health and Nutrition Examination Survey Data from 2001–2002 and 2011–2012. J. Dent. Hyg..

[6] Gevera P., Mouri H., Maronga G. (2019). Occurrence of Fluorosis in a Population Living in a High-Fluoride Groundwater Area: Nakuru Area in the Central Kenyan Rift Valley. Environ. Geochem. Health.

[7] Riordan P.J. (1993). Perceptions of Dental Fluorosis. J. Dent. Res..

[8] Centers for Disease Control and Prevention (1999). The Great Public Health Achivements—United States, 1900–1999. Morbidity and Mortality Weekly Report. https://www.cdc.gov/mmwr/pdf/wk/mm4812.pdf.

[9] Marthaler T.M. (2013). Salt fluoridation and oral health. Acta Med. Acad..

[10] O’Mullane D.M., Baez R.J., Jones S., Lennon M.A., Petersen P.E., Rugg-Gunn A.J., Whelton H., Whitford G.M. (2016). Fluoride and Oral Health. Community Dent. Health.

[11] García-Pérez Á., Irigoyen-Camacho M.E., Borges-Yáñez S.A., Zepeda-Zepeda M.A., Bolona-Gallardo I., Maupomé G. (2017). Impact of Caries and Dental Fluorosis on Oral Health-Related Quality of Life: A Cross-Sectional Study in Schoolchildren Receiving Water Naturally Fluoridated at above-Optimal Levels. Clin. Oral Investig..

[12] Pandya M., Diekwisch T.G.H. (2019). Enamel Biomimetics—Fiction or Future of Dentistry. Int. J. Oral Sci..

[13] Fraser S.J., Natarajan A.K., Clark A.S.S., Drummond B.K., Gordon K.C. (2015). A Raman Spectroscopic Study of Teeth Affected with Molar-Incisor Hypomineralisation. J. Raman Spectrosc..

[14] Penel G., Leroy G., Rey C., Bres E. (1998). MicroRaman Spectral Study of the PO_4_ and CO_3_ Vibrational Modes in Synthetic and Biological Apatites. Calcif. Tissue Int..

[15] Elliot J.C., Wilson R.M., Dowker S. (2002). Apatite Structures. Adv. X-ray Anal..

[16] Thomas D.B., Fordyce R.E., Frew R.D., Gordon K.C. (2007). A Rapid, Non-Destructive Method of Detecting Diagenetic Alteration in Fossil Bone Using Raman Spectroscopy. J. Raman Spectrosc..

[17] Thylstrup A., Fejerskov O. (1978). Clinical Appearance of Dental Fluorosis in Permanent Teeth in Relation to Histologic Changes. Community Dent. Oral Epidemiol..

[18] Zavala-Alonso V., Loyola-Rodríguez J.P., Terrones H., Patiño-Marín N., Martínez-Castañón G.A., Anusavice K. (2012). Analysis of the Molecular Structure of Human Enamel with Fluorosis Using Micro-Raman Spectroscopy. J. Oral Sci..

[19] Seredin P., Goloshchapov D., Ippolitov Y., Vongsvivut J. (2020). Development of a new approach to diagnosis of the early fluorosis forms by means of FTIR and Raman microspectroscopy. Sci. Rep..

[20] Auner G.W., Koya S.K., Huang C., Broadbent B., Trexler M., Auner Z., Elias A., Mehne K.C., Brusatori M.A. (2018). Applications of Raman Spectroscopy in Cancer Diagnosis. Cancer Metastasis Rev..

[21] Frost J., Ludeman L., Hillaby K., Gornall R., Lloyd G., Kendall C., Shore A.C., Stone N. (2017). Raman Spectroscopy and Multivariate Analysis for the Non Invasive Diagnosis of Clinically Inconclusive Vulval Lichen Sclerosus. Analyst.

[22] Neto L.P.M., e Silva L.F.D.C., Dos Santos L., Soto C.A.T., de Azevedo Canevari R., de Oliveira Santos A.B., Mello E.S., Pereira M.A., Cernea C.R., Brandao L.G. (2017). Micro-Raman Spectroscopic Study of Thyroid Tissues. Photodiagnosis Photodyn. Ther..

[23] Zheng X., Lv G., Zhang Y., Lv X., Gao Z., Tang J., Mo J. (2019). Rapid and Non-Invasive Screening of High Renin Hypertension Using Raman Spectroscopy and Different Classification Algorithms. Spectrochim. Acta Part A Mol. Biomol. Spectrosc..

[24] Sajda P. (2006). Machine Learning for Detection and Diagnosis of Disease. Annu. Rev. Biomed. Eng..

[25] Brauchle E., Schenke-Layland K. (2013). Raman Spectroscopy in Biomedicine—Non-Invasive in Vitro Analysis of Cells and Extracellular Matrix Components in Tissues. Biotechnol. J..

[26] Agrawal G., Samal S.K. (2018). Raman Spectroscopy for Advanced Polymeric Biomaterials. ACS Biomater. Sci. Eng..

[27] Salehi H., Collart-Dutilleul P.Y., Gergely C., Cuisinier F.J. (2015). Confocal Raman microscopy to monitor extracellular matrix during dental pulp stem cells differentiation. J. Biomed. Opt..

[28] Ramakrishnaiah R., Rehman G.U., Basavarajappa S., Al Khuraif A.A., Durgesh B.H., Khan A.S., Rehman I.U. (2015). Applications of Raman Spectroscopy in Dentistry: Analysis of Tooth Structure. Appl. Spectrosc. Rev..

[29] Senado de la Republica/Comisiones Unidas Punto de Acuerdo sobre el Consumo de Agua con Arsénico y Flúor. [Senate of the Republic/United Commissions. Point of Agreement on the Consumption of Water with Arsenic and Fluoride]. https://www.senado.gob.mx/64/gaceta_del_senado/documento/3616.

[30] Gugnani N., Pandit I.K., Srivastava N., Gupta M., Sharma M. (2011). International Caries Detection and Assessment System (ICDAS): A New Concept. Int. J. Clin. Pediatr. Dent..

[31] Pendrys D.G. (1999). The differential diagnosis of fluorosis. J. Public Health Dent..

[32] Secretaría de Salud PROYECTO de Norma Oficial Mexicana PROY-NOM-007-SSA3-2017, Para la Organización y Funcionamiento de los Laboratorios Clínicos. [Health Ministry. PROJECT of Official Mexican Standard PROY-NOM-007-SSA3-2017, For the Organization and Operation of Clinical Laboratories]. http://dof.gob.mx/nota_detalle.php?codigo=5511878&fecha=31/01/2018.

[33] Beleites C., Sergo V. HyperSpec: A Package to Handle Hyperspectral Data Sets in R. R Package Version 0.99-20180627. http://hyperspec.r_forge.r-project.org.

[34] Iqbal Z., Tomaselli V.P., Fahrenfeld O., Möller K.D., Ruszala F.A., Kostiner E. (1977). Polarized Raman Scattering and Low Frequency Infrared Study of Hydroxyapatite. Phys. Chem. Solids..

[35] De Asa P.N., Santos C., Pazo A., de Asa S., Cuscó R., Artús L. (1997). Vibrational Properties of Calcium Phosphate Compounds. 1. Raman Spectrum of beta-Tricalcium Phosphate. Chem Mater..

[36] González-Solís J.L., Martínez-Cano E., Magaña-López Y. (2015). Early Detection of Dental Fluorosis Using Raman Spectroscopy and Principal Component Analysis. Lasers Med. Sci..

[37] Buchwald T., Okulus Z., Szybowicz M. (2017). Raman Spectroscopy as a Tool of Early Dental Caries Detection-New Insights. J. Raman Spectrosc..

[38] Xu C., Reed R., Gorski J.P., Wang Y., Walker M.P. (2012). The Distribution of Carbonate in Enamel and Its Correlation with Structure and Mechanical Properties. J. Mater. Sci..

[39] Gong B., Oest M.E., Mann K.A., Damron T.A., Morris M.D. (2013). Raman Spectroscopy Demonstrates Prolonged Alteration of Bone Chemical Composition Following Extremity Localized Irradiation. Bone.

[40] DenBesten P., Li W. (2011). Chronic Fluoride Toxicity: Dental Fluorosis. Monogr. Oral Sci..

[41] Martinez-Mier E.A., Shone D.B., Buckley C.M., Ando M., Lippert F., Soto-Rojas A.E. (2016). Relationship between Enamel Fluorosis Severity and Fluoride Content. J. Dent..

[42] Suga S. (1989). Enamel Hypomineralization Viewed from the Pattern of Progressive Mineralization of Human and Monkey Developing Enamel. Adv. Dent. Res..

[43] Fejerskov O., Larsen M.J., Richards A., Baelum V. (1994). Dental Tissue Effects of Fluoride. Adv. Dent. Res..

[44] Sereda V., Ralbovsky N.M., Vasudev M.C., Naik R.R., Lednev I.K. (2016). Polarized Raman Spectroscopy for Determining the Orientation of Di-D-Phenylalanine Molecules in a Nanotube. J. Raman Spectrosc..

[45] Environmental Protection Agency (EPA) (2010). Fluoride: Dose-Response Analysis for Non-Cancer Effects. Office of Water, Health and Ecological Criteria Division, Washington, DC. EPA Doc. 820R10019. https://nepis.epa.gov/Exe/ZyPDF.cgi/P100N4S8.PDF?Dockey=P100N4S8.PDF.

[46] Spencer A.J., Do L.G., Mueller U., Baines J., Foley M., Peres M.A. (2018). Understanding Optimum Fluoride Intake from Population-Level Evidence. Adv. Dent. Res..

[47] Pramanik S., Saha D. (2017). The genetic influence in fluorosis. Environ. Toxicol. Pharmacol..

[48] Doty K.C., Lednev I.K. (2018). Raman spectroscopy for forensic purposes: Recent applications for serology and gunshot residue analysis. Trends Anal. Chem..

[49] Mohanta A. (2018). Dental Fluorosis—Revisited. Biomed. J. Sci. Tech. Res..

[50] Susheela A.K., Toteja G.S. (2018). Prevention & Control of Fluorosis & Linked Disorders: Developments in the 21st Century—Reaching out to Patients in the Community & Hospital Settings for Recovery. Indian J. Med. Res..

[51] Ding Y., Sun H., Han H., Wang W., Ji X., Liu X., Sun D. (2011). The Relationships between Low Levels of Urine Fluoride on Children’s Intelligence, Dental Fluorosis in Endemic Fluorosis Areas in Hulunbuir, Inner Mongolia, China. J. Hazard. Mater..

